# Beyond the Algorithm: A Critical and Evidence-Based Review of Artificial Intelligence in Chronic Pain Rehabilitation

**DOI:** 10.7759/cureus.113292

**Published:** 2026-07-24

**Authors:** Tijana Spasojevic, Aleksandar Knezevic

**Affiliations:** 1 Physical Medicine and Rehabilitation, Faculty of Medicine, University of Novi Sad, Novi Sad, SRB

**Keywords:** artificial intelligence, chronic pain, digital therapeutics, ethical design, explainable ai, implementation science, pain rehabilitation, patient-centered care, rehabilitation medicine, value-based care

## Abstract

Chronic pain presents a complex, multifactorial challenge in rehabilitation medicine, requiring nuanced, person-centered interventions. Artificial intelligence (AI) has emerged as a potential tool for personalizing diagnosis, predicting outcomes, and optimizing therapy, yet its integration into clinical practice remains fragmented and ethically underdeveloped. This study provides a critical, evidence-based synthesis of AI in chronic pain rehabilitation, drawing on findings from implementation studies, patient-experience research, and equity-by-design initiatives to assess where and how AI can responsibly enhance rehabilitation practices. A narrative review and critical synthesis were conducted using literature from PubMed, Scopus, and Web of Science published between 2015 and 2025. Five thematic domains emerged: spectrum of clinical maturity, patient experience and therapeutic alliance, algorithmic equity and bias, regulatory governance, and economic viability. AI applications ranged from low-touch mobile apps using sensor data to integrated Clinical Decision Support systems. Key barriers included algorithmic opacity, patient emotional burden, such as digital fatigue, biased datasets, and fragmented reimbursement models. Promising pathways included explainable and auditable AI, stakeholder co-creation, and value-based pricing. The findings emphasize that AI adoption success depends less on algorithmic sophistication and more on contextual fit, transparency, and ethical design. AI in pain rehabilitation must evolve from technological novelty to ethical necessity by aligning its development with clinical values and human well-being. Transparent systems, participatory development, dynamic oversight, and equitable access are essential for ensuring that AI enhances rather than amplifies disparities in healthcare.

## Introduction and background

Chronic pain affects over 20% of the adult population in the United States alone and is a leading contributor to disability worldwide [1]. Defined by its persistence beyond typical healing times and the significant emotional distress or functional disability it causes, chronic pain requires comprehensive management [2]. Because it is often resistant to standard pharmacological therapies, chronic pain necessitates multimodal and personalized rehabilitation strategies that span the biopsychosocial spectrum [3]. The integration of artificial Intelligence (AI) and machine learning (ML) offers the potential for personalized diagnosis, predictive outcome modeling, and optimized therapeutic delivery [4]. The convergence of human clinical expertise with computational precision - often termed "high-performance medicine" - has already shown vast potential across medical disciplines [5]. AI-driven predictive modeling is increasingly being utilized to forecast post-surgical pain and opioid utilization based on complex, high-dimensional clinical data [6]. Despite this promise, widespread clinical adoption in rehabilitation is lagging, constrained by ethical design limitations, fragmented implementation, and the need for robust evidence of real-world performance. This paper conducts a critical and evidence-based narrative review of AI in pain management. By synthesizing contemporary literature focusing on implementation science, patient-centered outcomes, and algorithmic equity, we evaluate AI through the lenses of clinical maturity, patient experience, algorithmic justice, regulatory preparedness, and economic scalability.

## Review

Materials and methods

We conducted a narrative review and critical synthesis of the literature. The interdisciplinary nature of the five thematic domains (clinical maturity, patient experience and therapeutic alliance, algorithmic equity and bias, regulatory governance, and economic viability) necessitated a broad synthesis rather than a narrow quantitative systematic review. Inclusion criteria focused on peer-reviewed articles, implementation studies, and ethical analyses addressing AI applications in chronic pain, musculoskeletal (MSK) rehabilitation, patient-reported outcomes, and algorithmic bias.

Results

Spectrum of Clinical Maturity

AI interventions in pain rehabilitation range from pilot applications to integrated digital therapeutics (DTx) [7]. For example, a recent retrospective cohort study evaluated medical motion, an AI-composed exercise program for personalized pain management. The study demonstrated significant real-world efficacy, showing a 1.78-point reduction on the numeric rating scale (NRS) for pain intensity and a 3.11-point improvement in patient well-being over eight weeks [8]. Similarly, advanced ML models utilizing large retrospective cohorts have achieved high accuracy in predicting individual pain relief and identifying key clinical indicators that affect chronic pain outcomes [9].

The collection of real-world subjective and objective markers via mobile health (mHealth) applications is also proving vital for understanding chronic pain in daily life [10]. However, AI adoption success is determined less by algorithmic sophistication and more by operational fit. Clinicians must prioritize systems that seamlessly integrate into existing clinical workflows and offer clear explainability to support shared decision-making, ensuring that the technology augments multidisciplinary care without alienating the user.

Patient Experience and Digital Fatigue

While mHealth tools show great potential in facilitating adherence to chronic musculoskeletal pain (CMP) management [11], the patient experience is often complex. Qualitative reviews of digital health in chronic disease management reveal a dichotomy: users appreciate the reassurance of continuous monitoring, but frequently experience "ambivalence" and "digital fatigue" [12]. The burden of continuous tracking, poor usability in certain applications, and the anxiety caused by an influx of health data can lead to therapy dropout. In fact, ML models are now being specifically deployed to predict which patients are at the highest risk of dropping out of chronic pain treatments so that providers can intervene earlier [13].

The Digital Therapeutic Alliance

A major concern in digital rehabilitation is the loss of the human-clinician bond. However, conversational agents (CAs) and chatbots are increasingly utilized for health interventions and cognitive-behavioral techniques [14]. Tools such as Wysa, an AI conversational agent, have shown surprising efficacy in building rapport. In studies evaluating the therapeutic alliance of users interacting with Wysa, individuals reported a mean working alliance bond score of 3.98 out of 5, demonstrating that users can form a perceived therapeutic alliance with an AI agent that is comparable to human-delivered psychotherapy [15]. While patients appreciate the consistent availability of the AI agent, it must be positioned as an auxiliary tool that supports, rather than replaces, embodied care.

Algorithmic Bias and Corrective Initiatives

Bias in AI is a documented reality in healthcare. A landmark study demonstrated that a widely used predictive algorithm in the US systematically underestimated the illness severity of Black patients compared to White patients [16]. The algorithm used healthcare costs as a proxy for health needs; because unequal access to care means less money is historically spent on Black patients, the algorithm falsely predicted they needed less care, reducing the percentage of Black patients identified for necessary extra care by more than half [16].

Corrective efforts are vital. The ability to mitigate algorithmic bias requires deliberate action across the AI life cycle, moving toward models of "shared responsibility" between developers, healthcare facilities, and regulatory bodies [17]. Furthermore, there is an urgent need to apply a health equity lens throughout the AI development cycle, intentionally designing algorithms that address the needs of historically excluded or marginalized populations [18]. Some researchers argue for a consequentialist framework that directly evaluates how algorithmic outputs impact distinct populations, rather than simply relying on equalized error rates [19].

Economic Viability and Systemic Barriers

AI development and maintenance costs are substantial. Current reimbursement schemes, which are typically episodic and procedure-based, are fundamentally incompatible with the continuous monitoring required for AI-driven rehabilitation. Value-based, remote models show considerable promise in this space. Fully remote digital care programs for musculoskeletal conditions have been shown to maintain high completion rates and significant clinical improvements, even for high-risk patient populations with complex comorbidities such as severe obesity [20].

Discussion

The promise of AI in chronic pain rehabilitation lies not only in computational power but in its alignment with the core values of person-centered care. Our findings suggest that the success of AI tools depends less on technical sophistication and more on contextual relevance, operational realism, and ethical design. Regulatory frameworks and ethical governance ecosystems must evolve to address the complexities of algorithmic transparency and dynamic risk, ensuring safe, inclusive innovation [21].

## Conclusions

AI in chronic pain rehabilitation should not be regarded as a simple technological add-on, but as the outcome of systemic design choices. In a field where empathy, adaptability, and complexity define clinical practice, AI must evolve in ways that are ethical, equitable, and human-centered. Key imperatives include participatory co-design with patients and clinicians to reduce digital fatigue; explainable and auditable systems that support, not supplant, clinical judgment; equity-by-design approaches to mitigate bias from the outset; moving away from flawed proxy variables; and sustainable financial models that reward long-term improvements in patient outcomes. When aligned with these principles, AI holds the capacity to transform the core mission of rehabilitation medicine.
